# Functional Analysis of Adipokinetic Hormone Signaling in *Bombyx mori*

**DOI:** 10.3390/cells9122667

**Published:** 2020-12-11

**Authors:** Hana Sehadova, Yoko Takasu, Anna Zaloudikova, Yu-Hsien Lin, Ivo Sauman, Hideki Sezutsu, Lenka Rouhova, Dalibor Kodrik, Michal Zurovec

**Affiliations:** 1 Biology Centre of the Czech Academy of Sciences, Institute of Entomology, Branisovska 31, 370 05 Ceske Budejovice, Czech Republic; sehadova@yahoo.com (H.S.); zalanna1@seznam.cz (A.Z.); r99632012@gmail.com (Y.-H.L.); sauman@entu.cas.cz (I.S.); rouhova.Lenka@seznam.cz (L.R.); kodrik@entu.cas.cz (D.K.); 2 Faculty of Science, University of South Bohemia, Branisovska 31, 370 05 Ceske Budejovice, Czech Republic; 3 National Institute of Agrobiological Sciences, 1-2 Owashi, Tsukuba, Ibaraki 305-8634, Japan; takasu@affrc.go.jp (Y.T.); hsezutsu@affrc.go.jp (H.S.)

**Keywords:** silkworm, targeted mutagenesis, TALEN, Bommo-AKH1, Bommo-AKH2, BMSK0010951, NM_001043584

## Abstract

Insect adipokinetic hormones (AKHs) are short peptides produced in the corpora cardiaca and are responsible for mobilizing energy stores from the fat body to the hemolymph. Three related peptides, AKH1, AKH2, and AKH/corazonin-related peptide (ACP) as well as three AKH receptors have been reported in *Bombyx mori*. AKH1 and AKH2 are specific for the AKHR1 receptor, whereas ACP interacts with the other two AKHRs. To assess the effect of the two silkworm AKHs and ACP in the regulation of energy homeostasis we examined the expression pattern of the three peptides and their receptors as well as their effect on the level of carbohydrates and lipids in the hemolymph. Our results support the hypothesis that only AKH1 and AKH2 peptides together with the AKHR1 receptor are involved in the maintenance of energy homeostasis. Because *Bombyx* AKHR1 (BmAKHR1) seems to be a true AKHR we generated its mutation. The *BmAKHR1* mutant larvae display significantly lower carbohydrate and lipid levels in the hemolymph and reduced sensitivity to starvation. Our study clarifies the role of BmAKHR1 in energy homeostasis.

## 1. Introduction

Insect adipokinetic hormones (AKHs) are well-studied insect neuropeptides belonging to the AKH/RPCH (red-pigment concentrating hormone) family [1]. They are produced by the corpora cardiaca, endocrine glands near the insect brain, and are involved in mobilizing energy stores and their associated biochemical, physiological, and behavioral processes [2]. Their physiological functions are mediated by adipokinetic hormone receptors (AKHRs), which belong to the G-protein-coupled receptor (GPCR) family, and have been identified in several insect species including *Drosophila melanogaster* and *Bombyx mori* [3,4]. Insect AKHRs are structurally and evolutionarily related to the gonadotrophin receptors (GnRH) of mammals [5,6,7]. Functionally, AKHR has a similar role to the mammalian glucagon receptor and is involved in the mobilization of carbohydrates and lipids [2]. Members of the AKH gene family undergo fast evolutionary changes and their number can vary from one in *D. melanogaster* [8] up to four (this probably includes AKH/corazonin-related peptide, ACP) similar AKH neuropeptides encoded by separate genes in insects such as the fall armyworm, *Spodoptera frugiperda* [9,10]. Ectopic AKH expression of *Drosophila* AKH was shown to induce both hypertrehalosemia and hyperlipemia in larval hemolymph [11]. In addition, the flies with ablated AKH neurons had reduced trehalose levels and showed strong resistance to starvation; however, the ablation might interfere with more signals than just AKH. *D. melanogaster AKH* and *AKHR* mutants were confirmed to be less sensitive to starvation and display increased glycogen and lipid accumulation in the fat body, irrespective of their nutritional status, as well as lower levels of carbohydrates in the hemolymph [12,13,14,15]. AKHR was suggested to signal both via Gαq and Gαs to activate cAMP and Ca^2+^-dependent second messenger pathways in the fat body of locusts, cockroaches, and beetles [16].

The first lepidopteran AKH peptide was identified from the tobacco hornworm, *Manduca sexta* [17], and the nonapeptides with the same sequence (pELTFTSSWG-amide) were later found in several other lepidopterans, including *B. mori* and *Heliothis zea* [18]. However this nonapeptide, called Manse-AKH or Bommo-AKH1, activates the *B. mori* AKH receptor with a lower affinity than *H. zea* hypertrehalosemic hormone [19], suggesting the presence of additional AKH-like peptides in the *B. mori* genome [20]. Two other distinct cDNAs were later identified in the silkworm based on homology searches [21,22]. The first one was later called Bommo-AKH2, whereas the second was initially identified as AKH and is now recognized as ACP. The ACP peptides form a distinct subfamily closely related to both AKH and corazonin [23]. Members of this subfamily are absent not only in *Drosophila* species, but also in *Apis mellifera* or *Acyrthosiphon pisum* and may have been lost several times during insect evolution [23].

The isolation of the first *B. mori* AKHR, BmAKHR1, was based on the sequence similarity with AKHR from *D. melanogaster* (DmAKHR); it has 405 amino acids showing 48% identity (68% conserved residues) [20]. Later on, two more genes encoding similar GPCR receptors *BNGR-A28* and *BNGR-A29*, also called *BmAKHR2a* and *-2b*, respectively, were identified in *B. mori* [24,25]. The proteins BNGR-A28 and -A29 contain 418 and 416 amino acid residues, respectively, and have 30.2% and 30.8% sequence identity with *B. mori* AKHR1. Transcripts encoding both BNGR-A28 and -A29 proteins were detected in larval testes primordia [25]. Ligand-receptor interactions in HEK293 cell culture showed that *B. mori* AKH1 and AKH2 both activated *B. mori* AKHR1, whereas ACP had higher affinity to the BNGR-A28 (AKHR2a) and BNGR-A29 (AKHR2b) [4,26]. The two receptors are also now considered as ACP receptors [23,25].

To learn more about AKH and ACP signaling in *B. mori* in vivo, we assessed the expression pattern of these peptides and their receptors. We also examined the effects of microinjecting the three peptides, AKH1, AKH2, and ACP, into larvae and adults on the levels of carbohydrates and lipids in the hemolymph. Our results support the hypothesis that ACP signaling mediated through BNGR-A28 (AKHR2a) and BNGR-A29 (AKHR2b) receptors differs from a bona fide AKH/AKHR system. Because BmAKHR1 seems to be a true ortholog of *Drosophila* AKHR, we studied its expression in more detail, generated a mutation in the *BmAKHR1* gene, and examined its effects on starvation resistance and metabolism.

## 2. Materials and Methods

### 2.1. Silkworm Strains and Crossing

A non-diapausing *B. mori* strain, *w1-pnd* (*white egg 1, pigmented and non-diapausing egg*), was used for site specific mutagenesis by microinjection of TALEN mRNAs. The strain was obtained at the Transgenic Silkworm Research Unit (National Agriculture and Food Research Organization, Tsukuba, Japan). Larvae of the polyvoltine line N4 were obtained from the Institute of Zoology, Bratislava (Slovakia) and were used for AKH microinjection experiments. The larvae were kept at low density on an artificial diet (Nihon Nosan Corporation, Yokohama, Japan) or mulberry leaves at 25 °C.

The crossing scheme for mutagenesis of the autosomal *BmAKHR1* gene was the same as in Takasu et al. [27]. The G1 heterozygotes were genotyped and their alleles were sequenced. Mutants carrying two different *BmAKHR1* alleles, one obtained by homology-directed repair and the other by non-homologous end joining, were used to construct homozygous lines (Figure 3). Verification of Mendelian ratios (Table S1) was performed by crossing heterozygotes and resulting first instar larvae were genotyped by PCR.

### 2.2. Synthetic Peptides

Synthetic adipokinetic peptide Bommo-AKH1 (pELTFTSSWGamide) [17]) was purchased from Bachem (Bubendorf, Switzerland), whereas Bommo-AKH2 (pELTFTPGWGQamide) [22]) and ACP (pEITFSRDWSGamide [21]) were purchased from Moravian Biotechnology Ltd., (Brno, Czech Republic).

### 2.3. TALEN Mutagenesis

The TALEN target sequence was chosen in the second exon of *BmAKHR1* encoding the first transmembrane domain; the spacer region contained a natural *Eco*RV site at its center to simplify mutation detection (Figure 3). DNA constructs carrying engineered TALE repeat arrays were then prepared by Golden Gate assembly [28] using the Golden Gate TALEN Kit from Addgene and inserted into the scaffold plasmid pBlue-TAL as previously described [27]. The constructs were purified using a HiSpeed plasmid midi kit (Qiagen, Hilden, Germany), linearized by digestion with *Xba*I, and treated with proteinase K (Nakarai, Kyoto, Japan). Capped and polyadenylated RNAs were then synthesized using an mMESSAGE mMACHINE T7 kit (Ambion, Carlsbad, CA, USA) as previously described in Takasu et al. [29]. A total of 3–5 nL of RNA solution was injected through the chorion into silkworm embryos at the syncytial preblastoderm stage (4–8 h after oviposition) as previously described [29]. We used single stranded oligonucleotide as a donor template for the embryo microinjection, enabling precise genome editing at the target site as in Takasu et al. [30].

Genomic DNA extracted from one leg was used for genotyping using 60 μL of DNAzol reagent (DNAzol Direct kit, Molecular Research Center, Cincinnati, OH, USA); the target region was then amplified using KOD FX Neo polymerase (TOYOBO, Osaka, Japan). The PCR products were analyzed on an agarose gel containing 1% LO3 (Takara-bio, Kusatsu, Japan) and 3% NuSieve GTG (Lonza, Basel, Switzerland). The primers used for PCR detection were as follows: AKHR-F, CAGAAGAACTGGTCGCATCTGCT; AKHR-R GGGACAAGTACTTATTTCAAGACCA. The donor nucleotide AKHR1-D was: ACGGTATACAGTGTGCTGATGGTGAATTCTTGAAACGCGCCAGCAGATTGGACATA.

### 2.4. RNA Isolation and Real-Time RT PCR

Adult *B. mori* moths were anaesthetized by CO_2_ and dissected in Ringer saline. Fifth-instar *B. mori* larvae were anaesthetized by sinking in water and dissected in Ringer saline. The tissues were frozen in liquid nitrogen and stored at −80 °C. Total RNA was extracted using RiboZol RNA Extraction Reagent (Amresco, Zottegem, Belgium) according the manufacturer’s instructions. The RNA was treated with a TURBO DNA-free Kit (Ambion, Naugatuck, CT, USA) to remove traces of genomic DNA. One microgram of RNA was used for cDNA synthesis using a RevertAid First Strand cDNA Synthesis Kit (Thermo Fisher Scientific, Waltham, MA, USA) with Oligo (dT)_18_ primer.

Relative gene expression was quantified by real-time-RT-PCR (qPCR) using HOT FIREPol EvaGreen qPCR Mix Plus (ROX, Solis BioDyne, Tartu, Estonia). Each qPCR reaction mixture contained 3 μL of 5× HOT FIREPol EvaGreen qPCR Mix Plus, 4 μL of 30 times diluted cDNA template, 250 nm forward and reverse primers, and water to make up the total volume of 15 μL. The primers are shown in Table S2. Reference primer sequences are described in Peng et al. [31]. The reaction was performed using a the CFX96 PCR light cycler (BioRad, Philadelphia, PA, USA) with the following program: initial activation at 95 °C for 12 min; followed by 40 cycles of denaturation at 95 °C for 15 s, annealing at 60 °C for 20 s, and elongation at 72 °C for 20 s. A final melt-curve step was included post-PCR (ramping from 65 °C to 95 °C by 0.5 °C every 5 s) to confirm the absence of any non-specific amplification. The efficiency of each primer pair was assessed by constructing a standard curve through four serial dilutions. Each q-RT-PCR experiment consisted of three to four independent biological replicates with three technical replicates for each parallel group. Each experiment was repeated two times. The reaction efficiency and Cq values were analyzed using Bio-Rad CFX Manager software. Relative gene expression was determined using the method described by Pfafl et al. [32].

### 2.5. In Situ Hybridization

A subcloned fragment of *B. mori AKHR1* was used as a template for antisense and sense (control) digoxigenin (DIG)-labeled riboprobe synthesis (MaxiScript labeling kit, Invitrogen, Waltham, MA, USA). Specific *AKHR1* oligonucleotide primers with attached phage SP6 or T7 polymerase promoter sequences (lowercase letters), respectively, were used for the riboprobe template synthesis: sense—BmAKHR1inFw caatttaggtgacactataGAAGGTCCGCCGCGCCATTATC, antisense—BmAKHR1inRev tgtaatacgactcactataATGCCGCTTCGACGCATCTTATC. The T7 polymerase was used for the antisense probe, whereas SP6 polymerase was used for the negative control sense probe. The riboprobe yield and DIG incorporation were assessed by spot blot. Dissected tissues of *B. mori* last instar larva were fixed in Bouin–Hollande solution without acetic acid but supplemented with mercuric chloride overnight at 4 °C [33]. In situ hybridization was performed as described elsewhere [34]. Briefly: tissue underwent dehydration, embedding in paraplast, sectioning to 7 µm, deparaffinization, and rehydration. The residual heavy metal ions in tissues were removed by treatment with Lugol’s iodine followed by 7.5% solution of sodium thiosulphate and distilled water. Samples were then treated with 0.2 N HCl for 20 min, washed with phosphate-buffered saline (PBS) supplemented with 0.3% Tween-20 (PBS-Tw), acetylated with 0.25% acetic anhydride in 0.1 M triethanolamine, and washed in PBS and 2× SSC. Prehybridization was performed in the hybridization buffer (IsHyb In situ Hybridization Kit, BioChain, Newark, CA, USA) at 60 °C for 3 h. The probes were diluted in hybridization solution to a final concentration of 0.005 ng/μL and then denatured for 10 min at 65 °C. Hybridization was allowed overnight at 60 °C. Stringency washes were performed twice with 2× SSC at 58 °C for 20 min, once with 1.5× SSC at 58 °C for 20 min, and twice with 0.2× SSC at 37 °C for 20 min each. After rinsing with PBS-Tw, the sections were blocked with 10% normal goat serum in PBS-Tw and incubated overnight at 4 °C with Fab fragments of sheep anti-DIG antibody conjugated to alkaline phosphatase (AP) (Boehringer Mannheim GmbH, Ingelheim, Germany), diluted at 1:500 in PBS-Tw. Following rinsing with PBS-Tw and detection buffer (IsHyb), the AP activity was detected with the nitroblue tetrazolium/bromo-chloro-indolyl phosphate substrate system (Perkin Elmer, Waltham, MA, USA). DIG-labeled sense probe was used in control experiments. In all cases, no signal was detected above background. Resulting samples were viewed under Olympus BX51 microscope equipped with PD80 CCD camera (Olympus).

### 2.6. Functional Assays

For the starvation survival assay of *B. mori* larvae, groups of fourth-instar larvae (day 1), including control and *BmAKHR1*(Δ7) mutants, were grown at a low density at 25 °C and 30–50% relative humidity and received only water. The dead individuals were counted at 12–24 h intervals until all larvae had died. The results were then expressed as the percentage of surviving larvae at different time points. The experiments were performed in triplicate.

For the quantification of free carbohydrates and lipids in the hemolymph, we used hemolymph samples taken from the cut at the top of abdominal leg of the last larval instar or abdominal cuticle in imagoes. A drop of hemolymph was leaked onto a piece of parafilm M and stored in an Eppendorf tube at −20 °C or used directly for an assay. Total free carbohydrates were determined by the colorimetric anthrone method [35]. The carbohydrate concentration was calculated from the calibration curve of standard glucose. The lipid content for each hemolymph sample was determined using an assay based on the sulpho-phosphovanilin test [36]. Optical densities at 546 nm, measured using a spectrophotometer (UV 1601, Shimadzu, Duisburg, Germany), were converted to µg lipids per µL hemolymph with the aid of a calibration graph based on known amounts of oleic acid.

For the mobilization of carbohydrates and lipids, we used *Bombyx mori* larvae of the last instar (2- to 3-day-old) or 1-day-old adult males (both polyvoltine line N4) fed on green leaf diet. They were injected with a dose of 50 pmol in 10% methanol (5 μL)/Ringer saline of each AKH peptide using insulin syringe without any anesthesia. Controls were injected with 10% methanol/Ringer saline only. After 1.5 h, the hemolymph was taken from the cut larval abdominal leg or from the cut abdominal cuticle in adults.

To use sensitized conditions in order to see peptide effects, we performed experiments with ligated larvae—larvae were neck ligated 24 h after last larval ecdysis and injected with hormones (50 pmol in 5 μL) or 10% methanol/Ringer saline 24 h later. The hemolymph was collected 1.5 h later and analyzed as described above.

### 2.7. Data Presentation and Statistical Analyses

The results were plotted using Prism v.6.0 (Graph Pad Software, San Diego, CA, USA). The numbers of replicates (n) are depicted in the figure legends. The use of statistical methods is specified in the relevant figure legends.

## 3. Results

### 3.1. In Vivo Expression of AKHs and AKHRs

**Figure 1:**
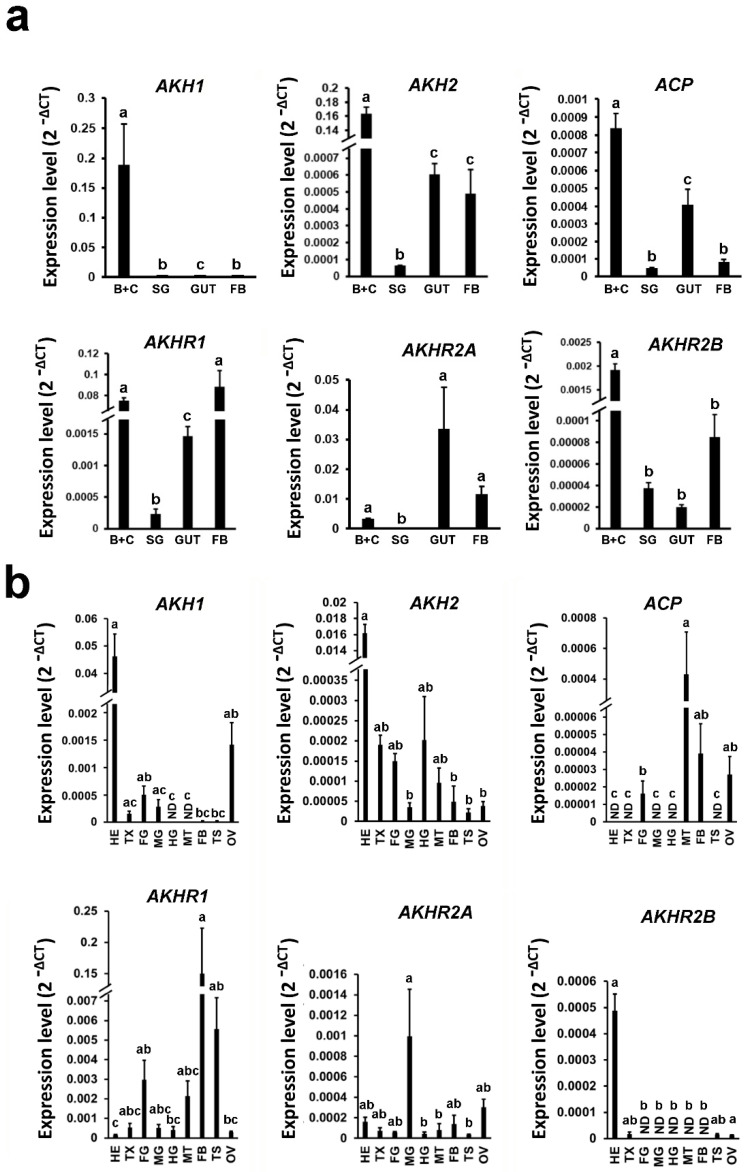
AKH and AKHR isoforms differ in the tissue specificity of their expression. RT-PCR analysis of *AKH* and *AKHR* mRNAs was performed using tissues from larvae (**a**) as well as adult moths (**b**). The adult RNA samples other than testes and ovary contained male and female tissues in equal proportions. The mRNA levels were measured relative to *BmActin,* and *BmTubulin* mRNAs. The samples contained pooled tissues from several individuals. Values represent means ± SD from three independent experiments. Data were analyzed by Kruskal–Wallis test followed by pairwise comparisons using Wilcoxon rank sum test. The significant differences are indicated by different letters (*p* < 0.5). HE—head; TX—thorax; FG—foregut; MG—midgut; HG—hind gut; MT—Malpighian tubules; FB—fat body; TS—testes; OV—ovary; B+C—brain with corpora cardiaca; ND—not detected.

In order to localize and characterize the tissue-specific distribution of mRNAs specific to *B. mori* AKHs and AKHRs, we analyzed their expression in several larval and imaginal tissues by RT-PCR (Figure 1a,b). The highest expression of AKH1 and AKH2 in both larvae and adults was observed in heads. ACP was found mainly in larval heads and guts, whereas in adults it was found in Malpighian tubules. Interestingly, the highest expression of BmAKHR1 in larvae was found in the fat body and heads, whereas the highest adult expression was detected in the fat body and testes. In contrast, the highest expression of BNGR-A28 (AKHR2a) was in the midgut of both larvae and adults. Finally, BNGR-A29 (AKHR2b) was mostly expressed in larval and adult heads (Figure 1a,b).

### 3.2. Effect of AKH1 and AKH2 on Hemolymph Lipid and Carbohydrate Levels

**Figure 2:**
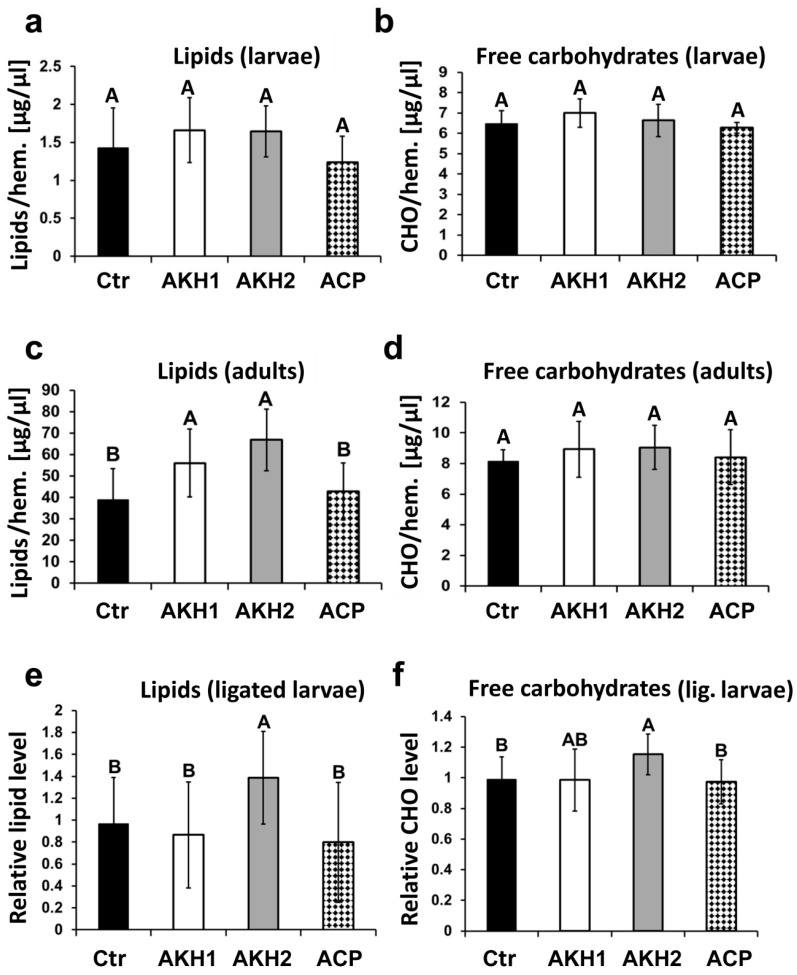
Effects of AKH peptides on the mobilization of carbohydrates and lipids. Fifth-instar larvae or adult moths were injected with 50 pmol of each peptide in 10% methanol/Ringer saline. Level of free lipids (**a**) and carbohydrates (**b**) in larval hemolymph; level of free lipids (**c**) and carbohydrates (**d**) in adult hemolymph; level of free lipids (**e**) and carbohydrates (**f**) in the hemolymph of ligated larvae. Ctr—saline-injected control group. Significant differences between groups on bar graphs are shown with different letters, *p* < 0.05, *n* = 16; *ANOVA* followed by *Bonferroni’s post hoc* test was used for statistical analysis. Error bars indicate standard deviation (SD).

To examine the effect of individual AKH peptides on the level of free lipids and carbohydrates in the hemolymph, we injected synthetic AKH1, AKH2, and ACP into fifth-instar larvae and adults. Although we did not observe any significant carbohydrate- or lipid-releasing effects in the larvae, injections of AKH1 and AKH2 into adults significantly increased lipid levels in their hemolymph (Figure 2). As shown in Figure 2c, we observed almost twice the amount of lipids in the hemolymph after injection. The effect of AKH2 was slightly stronger than AKH1. We did not observe any metabolic effects of ACP-like.

To further examine the relationship of AKHs and their receptors in larvae, we used sensitized conditions and injected the AKHs into the abdomen of neck ligated larvae. It was shown earlier that the trehalose hemolymph level of such larvae is reduced because of starvation and interruption (or substantial decrease) of AKH release into abdomen [37]. Our results showed that AKH2 was able to increase both carbohydrate, as well as lipid levels in the hemolymph of ligated larvae (Figure 2e,f). The ACP did not show significant effects in these assays, the AKH1 had only a marginal effect on carbohydrates.

### 3.3. Generation of BmAKHR1 Null Alleles

To investigate AKH signaling in silkworms in more detail, we used targeted mutagenesis to create a mutation in BmAKHR1 protein that serves as a receptor for both AKH1 and AKH2 peptides.

**Figure 3:**
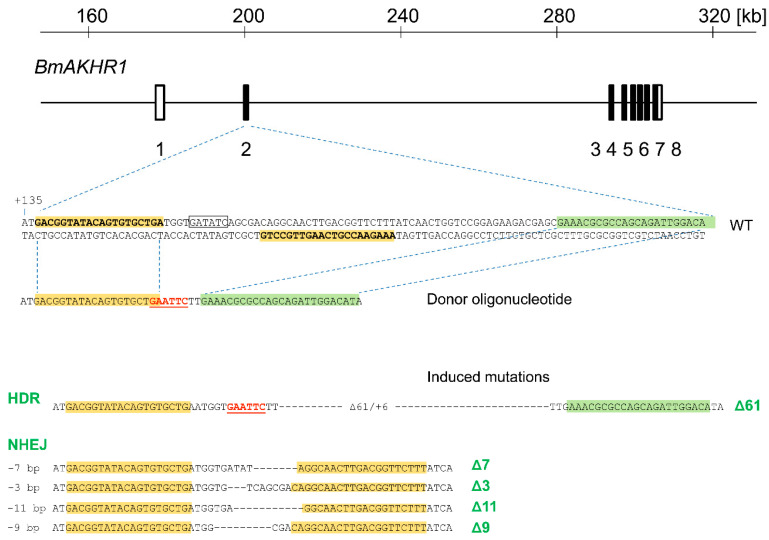
Mutagenesis of *BmAKHR1* using TALENs. Positions of TALEN target in the *BmAKHR1* gene; rectangles and numbers below represent exons. The numbers at the top of the panel indicate distances in kb. The sequence below the line indicates part of the second exon (WT) together with a donor oligonucleotide used for co-injection. The left and right *TALEN binding* sites are highlighted in yellow. *Eco*RV restriction site in wt sequence is boxed; *Eco*RI restriction site in the donor oligonucleotide is highlighted in red and underlined. Donor contains a 20 nt left arm homologous to the flanking sequence of the cleavage site and a 25 nt right arm (highlighted in green) homologous to the genomic sequence shifted downstream of the cleavage site, thus skipping the entire recognition site of right TALEN monomer. Sequences at the bottom indicate examples of induced mutations including HDR (a product of homology-directed repair) and several NHEJ (non-homologous end joining) events. Deletions in the sequence alignments are represented by dashes. The lengths of deletions are indicated to the left of the sequences, allele symbols to the right (green letters).

The genomic sequences of *BmAKHR1* were identified by BLAST in lepbase.org. The *BmAKHR1* gene spans more than 130 kb and has 8 exons (Figure 3). We mutagenized exon 2 using a TALEN pair based on the *pBlueTal* (NΔ152/C+63) framework [27]. Germline mutagenesis was performed as described in material and methods by microinjecting 91 eggs with TALEN mRNA. We also co-injected an oligonucleotide donor to create a 61 bp deletion and a 6 bp insertion by homology-directed repair (HDR). Mutant alleles were determined by PCR and sequencing. Fifty-seven G1 moths were genotyped, 6 of which carried a product of HDR. The remaining 51 did not contain *Eco*RI restriction site from donor oligonucleotide; therefore, most probably arose by nonhomologous end joining (NHEJ). The G1 moths, *BmAKHR1*(Δ61) carrying the 61 bp deletion and 6 bp insertion obtained by HDR and *BmAKHR1*(Δ7) containing the 7 bp deletion produced by NHEJ (Figure 3) were used in further experiments. Both mutants still encoded first (N-terminal) 51 amino acid residues of BmAKHR1 followed by out of frame sequences. Two homozygous mutant lines (Δ61 and Δ7) were established.

### 3.4. BmAKHR1 Mutant Larvae Are Resistant to Starvation and Have Low Levels of Carbohydrates and Lipids in the Hemolymph

Mutants in the *BmAKHR1* gene did not express any obvious phenotype, and homozygous adults were fertile. The cross between heterozygotes during the construction of homozygous mutant lines, however, appeared to produce homozygous mutant *BmAKHR1* individuals at a reduced frequency. Chi-square tests confirmed deviations from the expected outcomes of the segregation ratios for *BmAKHR1*(Δ61), suggesting partial embryonic lethality of this allele (Table S1). In contrast, the observed proportion of *BmAKHR1*(Δ7) genotypes was also lower, but it did not significantly deviate from the Mendelian frequency (Table S1). The phenotype of both mutant alleles may differ slightly from each other, but the differences might still be a result of random chance because both samples are small.

**Figure 4:**
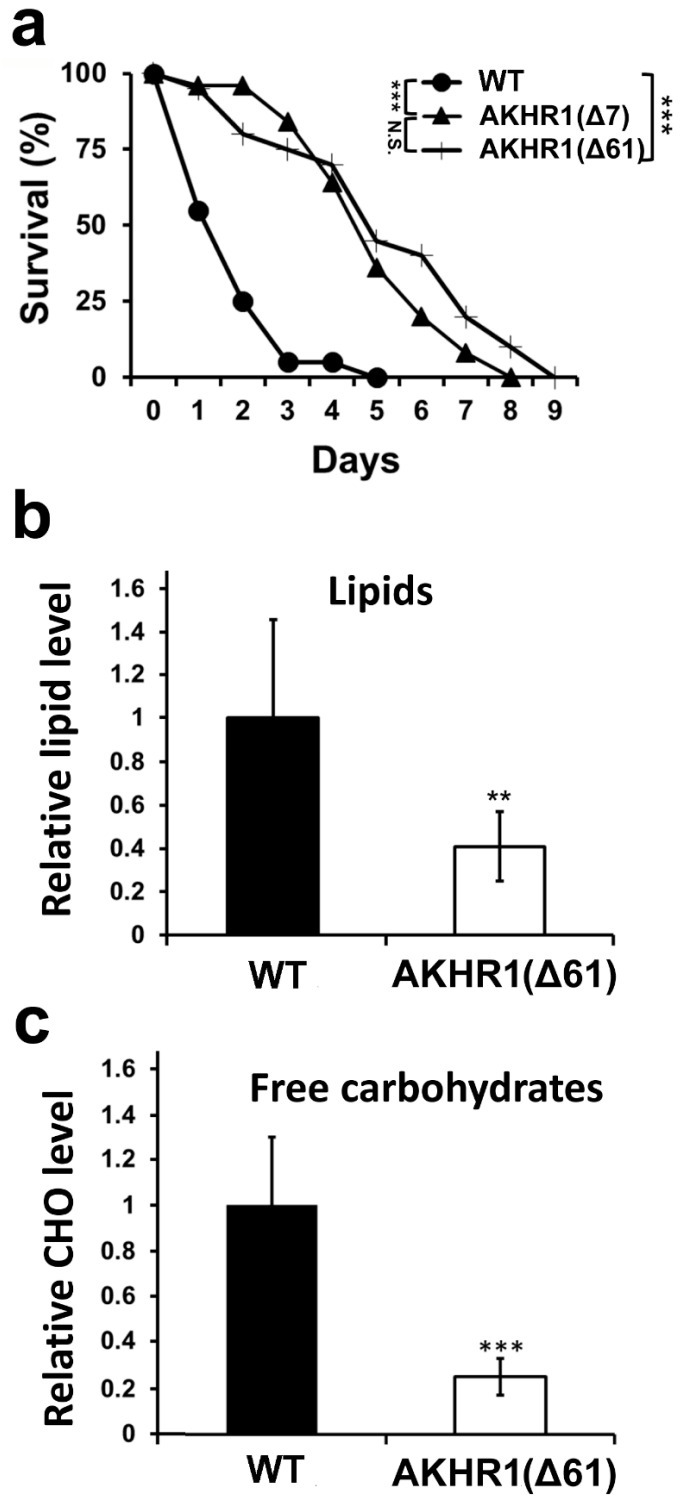
Characterization of *BmAKHR1* mutant. (**a**) Survival time course of fourth-instar control and *AKHR1* mutant larvae under starvation. Survival analysis was evaluated using a weighted log-rank test (Wilcoxon–Breslow–Gehan test). *** Significant difference, *p* < 0.01 compared to wild-type (WT); WT (*n* = 20), Δ61 (*n* = 25), Δ7 (*n* = 20). Levels of total free carbohydrates—CHO (**b**) and lipids (**c**) in larval hemolymph. Statistically significant differences between the control and other lines were evaluated using a two-tailed t-test. ** Significant difference, *p* < 0.01, *** *p* < 0.001; error bars indicate standard deviation (SD); *n* = 8.

Because the key activity of AKH signaling in insects is the mobilization of energy stores, we examined the resistance of *BmAKHR1*(Δ61) and *BmAKHR1*(Δ7) mutant individuals to starvation. We used fourth instar larvae, which have lower energy supplies and are easier to test than last instar larvae. As shown in Figure 4a, there was a striking difference between mutants and wild-type controls, with mutant larvae surviving more than twice as long as controls. The difference in survival between both mutant lines was not significant. These results show that *BmAKHR1* mutants are more starvation resistant than controls.

To obtain a more complete picture of the regulation of *BmAKHR1* mutant energy stores, we measured the levels of carbohydrates in the hemolymph of wild-type and mutant last-instar larvae. As shown in Figure 4b, the levels of free carbohydrates in the hemolymph of *BmAKHR1*(Δ61) mutants were significantly (more than 3 times) lower than control wild-type, showing a strong hypoglycemia.

Finally, the measurement of hemolymph lipids in mutant larvae *BmAKHR1*(Δ61) also showed a substantial reduction (Figure 4c) compared with wild-type larvae. The mutant larvae displayed similar changes: 2.5-fold lower concentrations of the free lipids in the hemolymph. These results suggest that mutants in *AKHR1* have impaired control of carbohydrate and lipid homeostasis.

### 3.5. Expression of BmAKHR1 In Vivo

**Figure 5:**
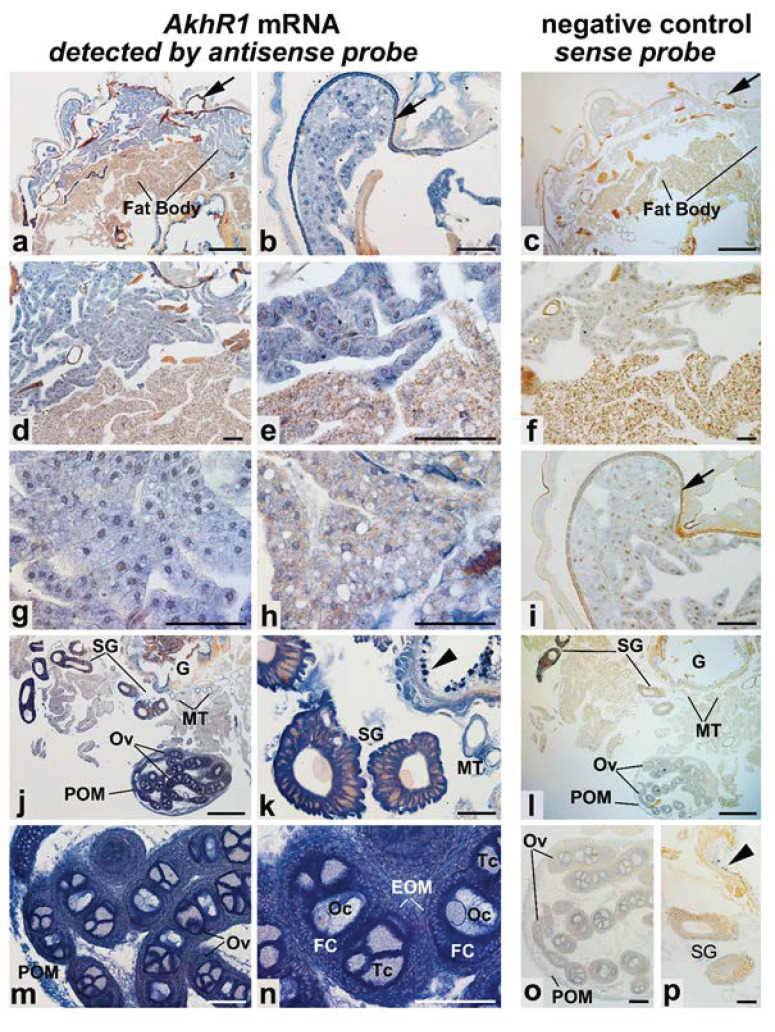
In situ expression of *BmAKHR1* mRNA in *Bombyx mori* larval tissues. (**a**) *AKHR1* mRNA expression in two different types of the fat body (peripheral and perivisceral fat body) and in the epidermis. (**b**) A detailed view of the epidermis and adjoining fat body. (**c**) The sense probe did not label any tissues in the parallel section to the one shown in A. (**d**,**e**) Different magnification images of a transition between the peripheral and visceral fat body. (**f**) Sense probe labeling on a control section parallel to the section in D. (**g**,**h**) A detailed view of the positive signal in the peripheral and the perivisceral fat body, respectively. (**i**) A control section parallel to that in B did not show any positive signal with sense probe. (**j**,**l**) Two parallel sections of the ventral body part show staining with antisense and sense probe, respectively. (**k**) Higher magnification view of the positive signal in the salivary glands (SG) and Malpighian tubules (MT). (**m**,**n**) High magnifications of the extensive signal in the ovarian follicles of the developing ovary. (**l**,**o**,**p**) Control preparation of the ovary and salivary glands in the close location to the gut (G), respectively. Abbreviations: *EOM*, epithelial ovarian membrane; *FC*, follicle cells; *G*, gut; *MT*, Malpighian tubules; *Oc*, oocyte; *Ov*, ovarioles; *POM*, peritoneal ovarian membrane; *SG*, salivary glands; *Tc*, trophocyte. Arrows depict the epidermis and arrowheads the midgut epithelium. Scale bar a, c, j, l, 500 μm, b, d–i, k, m–p, 100 μm.

Our data suggest that BmAKHR1 plays a key role in energy mobilization from the fat body. In order to further characterize BmAKHR1, we also examined in detail its expression in larval tissues (fifth-instar larvae) using in situ hybridization on the serial paraplast cross sections from the medioposterior segments. As shown in Figure 5, in situ hybridization with antisense probe revealed a widespread distribution of the positive signal in the variety of the *B. mori* larval tissues. Intense staining was found in the peripheral fat body (Figure 5a,b,d,e,g). Positive signal in the visceral fat body layer is partially hidden by orange colored droplets (Figure 5a,d,e,h). The epidermis also showed very intense staining (Figure 5a,b). In addition to the fat body and epidermis, a large amount of *AKHR1* mRNA was found in the salivary glands (Figure 5j,k), Malpighian tubules (Figure 5j,k), in the midgut epithelium (Figure 5j,k), and in the ovaries (Figure 5j,m,n). In the developing ovaries, the *AKHR1 mRNA* is located in the peritoneal ovarian membrane, in the epithelial ovarian membrane, and in the cells of developing ovarian follicles—follicle cells, trophocytes (nurse cells), and oocytes. Negative control with sense probe in parallel sections did not show any signal above background (right column of Figure 5).

## 4. Discussion

Previous studies have identified three AKH-like peptides and three AKHR-like receptors in *B. mori* and their functional activities were determined in cell culture in vitro. To shed more light on their function in vivo, we assessed the expression pattern of these peptides and their receptors in silkworm larvae and adults. We also examined the effects of AKH1, AKH2, and ACP microinjection on the mobilization of hemolymph carbohydrates and lipids. In addition, we prepared and characterized a mutant in *BmAKHR1* gene using targeted mutagenesis.

Earlier studies postulated that AKH is mainly produced by the corpora cardiaca, whereas the highest expression of AKHRs has been described in the fat body of different insects, including *D. melanogaster*, *M. sexta, Locusta migratoria*, or *A. pisum* [38,39,40]. Consistently, we confirmed the highest expression of BmAKHR1 is in the fat body, as expected for the AKH signaling pathway. High expression was also observed in tissues with high energy demand, including follicular cells and trophocytes of the ovary and gut. The expression of *BmAKHR1* in the silkworm larval fat body, midgut, and muscles was also previously observed by Yamanaka et al. [25]. Moreover, both AKH1 and AKH2 were able to stimulate lipid mobilization to the *B. mori* hemolymph of adults; AKH2 is also able to stimulate lipid mobilization in ligated silkworm larvae. In addition, AKH2 also stimulated the level of free carbohydrates in ligated larvae, further supporting the role of these two peptides as bona fide AKH hormones working through BmAKHR1. The microinjection of AKH2 had slightly stronger effects than AKH1. Interestingly, AKH2 has been previously suggested as having a higher affinity to BmAKHR1 than AKH1 [21]. Activation of lipolysis induced by AKH microinjection was previously observed in *M. sexta* [41]. The increase in hemolymph carbohydrate levels in our experiments in response to AKH signaling seemed weaker than the effects on lipids. Conversely, the expression pattern of BNGR-A29 and BNGR-A28 receptors does not support their direct involvement in energy metabolism. Additionally, the expression pattern of ACP peptide as well as the lack of its activity in the substrate mobilization bioassay confirms that ACP together with AKHR2A and -B are part of the separate ACP signaling pathway distinct from both AKHs and corazonins; their function is as yet unknown except that they do not mobilize lipids and carbohydrates. Consistently, the related ACP peptide from *L. migratoria*, called Lom-HrTH, showed no biological activity usually associated with AKHs [9].

The phenotype of our *BmAKHR1* mutant further supported the hypothesis that this receptor is involved in the mobilization of both lipids and carbohydrates. The lack of BmAKHR1 signaling leads to lower levels of substrates being released into the larval hemolymph. This phenotype is similar to the phenotypes of *D. melanogaster AKH* or *DmAKHR* mutants observed in earlier studies [13,15]. The starvation assay was performed on fourth instar larvae (they have similar levels of carbohydrates in the hemolymph as fifth instar larvae—see Figure S1) and showed lower sensitivity to starvation of individuals lacking AKHR1 signaling, which is consistent with previously published data on starvation of *D. melanogaster AKHR* mutant larvae [12,13,14,15].

We detected the highest *BmAKHR1* gene expression in the larval and adult fat body. It is in line with the earlier results on AKHRs in other species, including *D. melanogaster, M. sexta*, and *L. migratoria* [38,39]. The expression of BmAKHR1 in peripheral fat body tissue rather than at the visceral compartment suggests that AKHR signaling regulates metabolism in the last instar larvae, preferably in the peripheral fat body (synthesis site) rather than perivisceral fat body (major storage site). Peripheral fat body is a major source of energy for metamorphosis [42,43]. High *BmAKHR1* expression in the ovaries, salivary glands, and Malpighian tubules suggests that this pathway is active in tissues with high energy requirements. In several insect species, AKH was shown to inhibit the egg production mostly by curbing the vitellogenin synthesis [44]. It will be interesting to see whether the reduced proportion of mutants obtained in the cross of *BmAKHR1*(Δ61) heterozygotes (Table S1) is related to the absence of AKHR1 expression in mutant eggs.

Recent progress in reverse genetic methods allows genetic analyses to be performed in insects other than *D. melanogaster*. Targeted mutagenesis of *BmAKHR1* showed that mutant silkworm larvae have significantly lower carbohydrate and lipid levels in the hemolymph and display reduced sensitivity to starvation, a phenotype similar to *D. melanogaster AKHR* mutant, which has slower metabolic rate than wt [14,15]. Knockdown of AKH signaling was reported earlier to affect hemolymph lipid changes in the cricket *Gryllus bimaculatus* [45] or tenebrionid beetle *Zophobas atratus* [46]. Our results are in line with the notion that *Bombyx* AKH1 and AKH2 work as bona fide AKH hormones through BmAKHR1 receptor and are involved in the mobilization of energy stores in *B. mori*. They also show the conservation of AKH signaling between Diptera and Lepidoptera.

*B. mori* is one of a few known insect species that have more than one AKH. This phenomenon is well known, but its biological meaning is unclear. Some authors have indicated a partial functional specialization of AKHs in the insect body. For example, Kodrik and Goldsworthy [47] found a different efficacy of three *L. migratoria* AKHs in suppressing RNA synthesis in the fat body, and Candy [48] recorded differences in the release of two AKHs into the hemolymph of *S. gregaria* after insecticide treatment; however, this does not fully solve the problem. In *B. mori*, the situation is further complicated by the presence of ACP peptide, the function of which has not yet been satisfactorily explained. More studies are needed to clarify biological background of this phenomenon.

## Supplementary Materials

The following are available online at https://www.mdpi.com/2073-4409/9/12/2667/s1, Figure S1: Level of free carbohydrates in silkworm larval hemolymph of fourth and fifth instars. Table S1: Contingency tables comparing observed and expected results (proportion of mutants). Table S2: Primer list for qPCR.
