# Supplementary material for: Functional Analysis of Adipokinetic Hormone Signaling in Bombyx mori
Source: Cells. 2020 Dec 11;9(12):2667. doi: 10.3390/cells9122667 (PMC7764666; doi:10.3390/cells9122667)
Supplement: Supplementary file 1 [file cells-09-02667-s001.pdf]

Supplementary Information

Functional analysis of adipokinetic hormone signaling in *Bombyx mori*

Yoko Takasu[1], Anna Zaloudikova[2], Yu-Hsien Lin[2,3], Hana Sehadova[2,3], Ivo Sauman[2,3], Hideki Sezutsu[1], Lenka Rouhová[2,3], Dalibor Kodrik[2,3] and Michal Zurovec*[2,3]

[1]National Institute of Agrobiological Sciences, 1-2 Owashi, Tsukuba, Ibaraki 305-8634, Japan.
[2]Biology Centre of the Czech Academy of Sciences, Institute of Entomology, Branisovska 31, 370 05 Ceske Budejovice, Czech Republic
[3]Faculty of Science, University of South Bohemia, Branisovska 31, 370 05 Ceske Budejovice, Czech Republic

Contents:

Supplementary Figure 1: Level of free carbohydrates in larval hemolymph of 4th and fifth instars fed with mulberry leaves and artificial diet.

Supplementary Table 1: Contingency tables comparing observed and expected results

Supplementary Table 2: Primer list

Supplementary Figure 1: Level of free carbohydrates in larval hemolymph of fourth and fifth instars in larvae fed with mulberry leaves and artificial diet. Two tail t-test * P<0.05; *** P<0.001; error bars indicate standard deviation (SD); day 1/4th instar (early 4th); day 3-4/4th instar (late 4th); day 2-3/5th instar (early 5th); day 5/5th instar (late 5th).

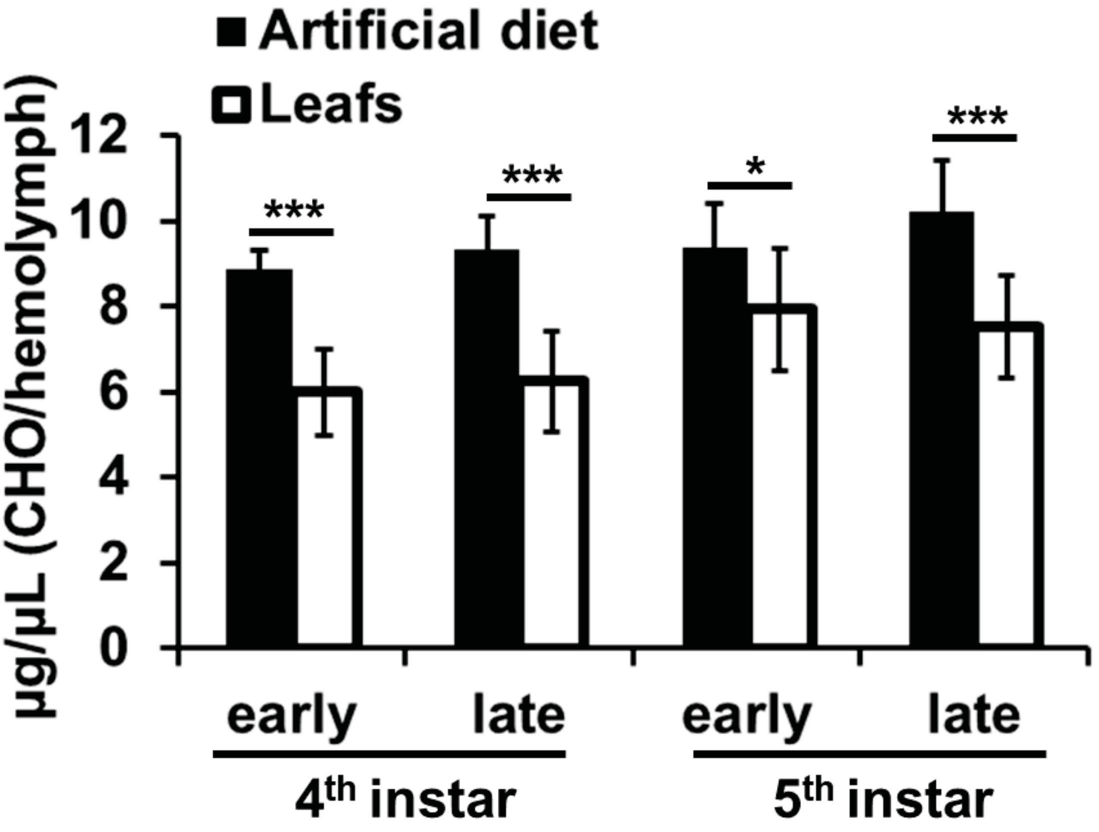

Supplementary Table 1: Contingency tables comparing observed and expected results of a (A) monohybrid cross between heterozygous individuals *BmAkhR1(Δ61)/+*.

Category	Observed	Expected	Deg. freedom	Chi square	P
Mut Δ61	8	13.25	2	8.321	0.016
Heterozygote	23	26.5			
wt	22	13.25			
**Total**	53				

The calculated value of 8.321 exceeds the probability of 0.05, but does not reach the value of 9.21 required for the probability of 0.01. At the 0.05 level the observed Δ61 genotypes deviate from the Mendelian ratio of 1: 2: 1.

(B) outcomes of a testcross between heterozygous individuals *BmAkhR1(Δ7)/+*

Category	Observed	Expected	Deg. freedom	Chi square	P
Mut Δ7	8	9.75	2	1.513	0.469
Heterozygote	18	19.5			
wt	13	9.75			
**Total**	39				

The calculated value of 1.513 does not exceed the probability of 0.05; the observed Δ7 genotypes do not deviate from the Mendelian ratio of 1: 2: 1.

Table S2. Primer list for qPCR

Gene	Primer (5'-3')
AKH1_F	GCCGAAGCCCAACTCACT
AHK1_R	AGGCATTTTGTCGGTGTTTCTGTT
AKH2_F	GCGCTTTGGTACTTGTGTTG
AKH2_R	ACCGGCACGCTAGAAACTT
AKH3_F	CCGACCGTATTTCAGTTCGT
AKH3_R	GTGGTCCGCTAGAGCAAATC
AKHR_F	AGGTTACAACCAATGCGTGTCTTAC
AKHR_R	GCTCTCCTGATGATCTCGAATAGC
AKHR2A_F	CACTATTGTCACTGTCTTCGCTTGTTG
AKHR2A_R	TCTAAGGTCCACGGTGTAGGAGCC
AKHR2B_F	AACCATTGTGTCAGTCTTCGCTTTG
AKHR2B_R	ATGTCCAGGGTGTAGGAGCCGTAG
Actin-3_F	CGGCTACTCGTTCACTACC
Actin-3_R	CCGTCGGGAAGTTCGTAAG
α-tubulin_F	CTCCCTCCTCCATACCCT
α-tubulin_R	ATCAACTACCAGCCACCC
